# Drug Repurposing for Atopic Dermatitis by Integration of Gene Networking and Genomic Information

**DOI:** 10.3389/fimmu.2021.724277

**Published:** 2021-10-13

**Authors:** Wirawan Adikusuma, Lalu Muhammad Irham, Wan-Hsuan Chou, Henry Sung-Ching Wong, Eko Mugiyanto, Jafit Ting, Dyah Aryani Perwitasari, Wei-Pin Chang, Wei-Chiao Chang

**Affiliations:** ^1^ Department of Clinical Pharmacy, School of Pharmacy, Taipei Medical University, Taipei, Taiwan; ^2^ Department of Pharmacy, Faculty of Health Science, University of Muhammadiyah Mataram, Mataram, Indonesia; ^3^ Faculty of Pharmacy, University of Ahmad Dahlan, Yogyakarta, Indonesia; ^4^ Ph. D. Program in the Clinical Drug Development of Herbal Medicines, College of Pharmacy, Taipei Medical University, Taipei, Taiwan; ^5^ Department of Pharmacy, Faculty of Health Science, University of Muhammadiyah Pekajangan Pekalongan, Pekalongan, Indonesia; ^6^ School of Health Care Administration, College of Management, Taipei Medical University, Taipei, Taiwan; ^7^ Taipei Medical University (TMU) Research Center of Cancer Translational Medicine, Taipei, Taiwan; ^8^ Department of Pharmacy, Wan Fang Hospital, Taipei Medical University, Taipei, Taiwan; ^9^ Integrative Research Center for Critical Care, Wan Fang Hospital, Taipei Medical University, Taipei, Taiwan; ^ 10^ Department of Pharmacology, National Defense Medical Center, Taipei, Taiwan

**Keywords:** atopic dermatitis, bioinformatics, drug repurposing, functional annotation, genetic

## Abstract

Atopic Dermatitis (AD) is a chronic and relapsing skin disease. The medications for treating AD are still limited, most of them are topical corticosteroid creams or antibiotics. The current study attempted to discover potential AD treatments by integrating a gene network and genomic analytic approaches. Herein, the Single Nucleotide Polymorphism (SNPs) associated with AD were extracted from the GWAS catalog. We identified 70 AD-associated loci, and then 94 AD risk genes were found by extending to proximal SNPs based on *r^2^* > 0.8 in Asian populations using HaploReg v4.1. Next, we prioritized the AD risk genes using *in silico* pipelines of bioinformatic analysis based on six functional annotations to identify biological AD risk genes. Finally, we expanded them according to the molecular interactions using the STRING database to find the drug target genes. Our analysis showed 27 biological AD risk genes, and they were mapped to 76 drug target genes. According to DrugBank and Therapeutic Target Database, 25 drug target genes overlapping with 53 drugs were identified. Importantly, dupilumab, which is approved for AD, was successfully identified in this bioinformatic analysis. Furthermore, ten drugs were found to be potentially useful for AD with clinical or preclinical evidence. In particular, we identified filgotinub and fedratinib, targeting gene JAK1, as potential drugs for AD. Furthermore, four monoclonal antibody drugs (lebrikizumab, tralokinumab, tocilizumab, and canakinumab) were successfully identified as promising for AD repurposing. In sum, the results showed the feasibility of gene networking and genomic information as a potential drug discovery resource.

## Introduction

Atopic Dermatitis (AD), also called atopic eczema, is a common chronic or relapsing skin inflammatory disease with characteristic acute flare-ups of eczematous pruritic lesions and dry skin (1, 2). AD is the most common skin disease in children; the prevalence is 15%~20% in children and 1%~3% in adults (1). Approximately 80 percent of the young patients remain symptomatic in adulthood, and they are mostly presented with lesions affecting the flexures, head, and neck (3). Both genetic and environmental factors have been reported to be involved in the pathogenesis of AD (4, 5). AD is a multifactorial disease with immunological processes, including type 1 IgE dysfunction, cell-mediated immune response defects, and barrier dysfunction changes (5). Genome-wide association studies (GWAS) and genetic association studies have reported many single nucleotide polymorphisms (SNPs) that were associated with AD pathogenesis, including Toll-like receptors (*TLRs*), *IRF2*, *IL-4, IL-13, IL-25, IL-31, IL-33, IL1RL1/IL18R1/IL18RAP, STAT6, ORAI1* and *TSLP* (6–11). Findings from GWAS have shown the complex role of multiple loci in human AD susceptibility. New insights concerning the genetic, immunological, and environmental impacts of AD provide novel therapeutic strategies against AD (12).

Management of AD is dependent on the severity of the disease. Epidermal obstructions play an essential role in the onset of AD (13). Two drugs have been approved by the U.S. Food and Drug Administration (FDA), which have increased treatment options for eczema. First of all, 2% Crisaborole ointment is approved for mild to moderate AD in children (14, 15). Furthermore, dupilumab is approved for adults with moderate to severe AD (16). However, these drugs are effective in only about 20% of moderate to severe AD patients (17). Therefore, developing new drugs for AD is urgent. Traditional drug discovery requires a long process (10~17 years) from an idea in the laboratory to a marketed drug with less than 10% overall probability of success (18). There are notable advantages of drug repurposing over the traditional drug discovery process; for instance, repurposed drugs have already passed clinical trials for their first indications, which is more time and cost efficient for drug development (19, 20). In addition, drug repurposing is able to reduce safety and pharmacokinetic uncertainties (21). An example of clinically successful drug repurposing is ketoconazole for Cushing syndrome, initially used for fungal infection. Another example is raloxifene used initially for osteoporosis and is now successfully repurposed for breast cancer (22).

In 2014, Okada et al. proposed bioinformatics drug discovery methodologies for rheumatoid arthritis (RA). Data from GWAS meta-analysis in RA was applied to identify risk loci for functional annotations and drug repurposing. Results were further applied to investigate potential candidate drug targets for RA (23). In the current study, we aimed to implement this bioinformatics strategy and identify AD’s biological candidate genes through an integrative gene network. Six functional annotations (missense mutations, cis-expression quantitative trait loci (cis-eQTL), a molecular pathway analysis, proteinprotein interactions (PPIs), genetic overlap with a knockout mouse phenotype, and primary immunodeficiencies (PIDs)) were used to discover biological AD risk genes.

## Methods

### Study Design

**Figure 1:**
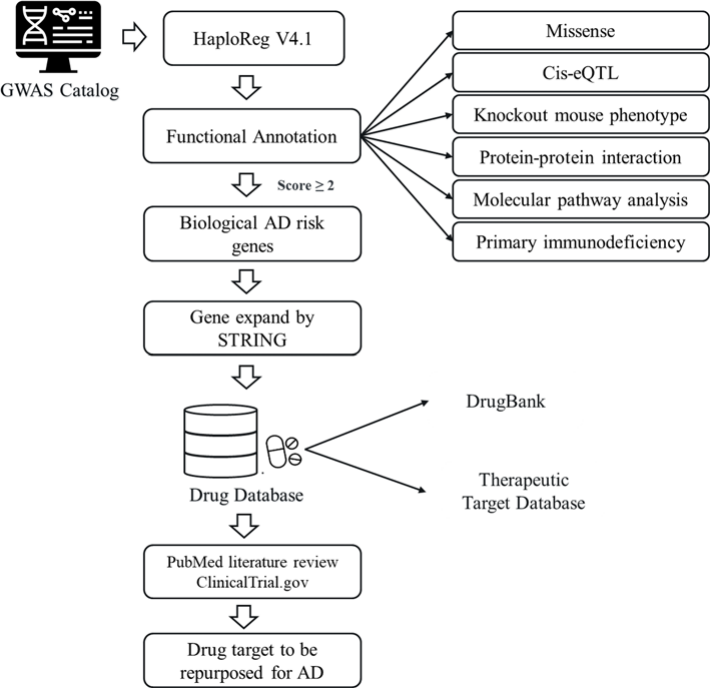
An overview of drug repurposing for atopic dermatitis (AD). The study design utilizing by GWAS Catalog and various databases: DrugBank database, Therapeutic Target Database (TTD), ClinicalTrial.gov, and PubMed.

A descriptive scheme of the current drug repurposing study for AD was shown in **Figure 1**. The SNPs with significant association with AD (*p* < 10^-5^) were queried from the National Human Genome Research Institute (NHGRI) GWAS catalog database (http://www.ebi.ac.uk/gwas) (24) on January 7, 2019. The SNPs adjacent to the AD associated SNPs were included based on Linkage Disequilibrium (LD) characteristic to define the AD risk SNPs. It was conducted using HaploReg (v4.1) (25) with the criterion of *r^2^* ≥ 0.8 in Asian (ASN) populations from the 1000 Genome Project Phase I data. The AD risk SNPs were classified into missense (or nonsense), synonymous or non-coding (with or without *cis*-eQTL) SNPs. In addition, the genes encoded by the AD risk SNPs will be used for further analyses. The AD risk genes were subsequently prioritized based on six functional annotation criteria. Accordingly, those genes with one functional annotation obtained one point (score), and those genes which met criteria with a score of ≥ 2 were defined as “biological AD risk genes”. In our analyses, we set the threshold of biological score ≥ 2 to find a much higher number of genes as the biological AD risk genes. The STRING database (26) was used to expand biological risk genes. The extended list was further defined as drug target genes. We mapped those drug target genes to DrugBank (27) and Therapeutic Target Database (TTD) (28). The drugs identified were examined for their clinical status, according to ClinicalTrials.gov.

### Functional Annotation of AD Risk Genes

Six biological functional annotations were used to build a scoring system representing the most probable candidate genes as AD targets. The six biological functional annotations were as follows: (i) missense or nonsense variants: We used RStudio v3.4.3 and the HaploR package (29), which contain annotations of functional consequences from a database of SNPs (dbSNPs) to perform this biological functional annotation. Missense or nonsense variants can be one method for functional annotation because the change in the amino acid sequence may affect protein function (30). If a gene had any missense or nonsense AD risk SNP, it was assigned with one point; (ii) *cis*-eQTLs: A SNP with cis-eQTL effect is associated with the expression of the gene at where the SNP is located. The polymorphism is associated with the change in gene expression in the target tissue, resulting in biological implications. If a gene had any AD risk SNP with *cis-*eQTL effect in the whole blood, it was assigned one point; (iii) KO mice: The gene from human Ensemble ID was converted to mouse Ensemble ID using BioMart to query the mouse phenotype (31). The data source was Mammalian Phenotype Ontology (MP), with information on mice and other mammal phenotypes (32). The gene set, with an FDR of < 0.05 in the enrichment analysis, were considered significant. (iv) gene ontology: The data source was the biological process category of Gene Ontology (GO). The significance of an FDR < 0.05 was set (33); (v) molecular pathways: Enrichment analysis was performed on molecular pathways using the Kyoto Encyclopedia of Genes and Genomes (KEGG), an online biochemical route database. The genes enriched in the KEGG pathway (FDR < 0.05) were assigned with one point (33); and (vi) PID: The PID was the final annotation to prioritize the AD risk genes. PID genes were collected by the IUIS until 2013 (34). Enrichment analysis on the data was conducted using a hypergeometric test; a p-value < 0.05 was used in this step as the significance criterion.

### Expansion Network by STRING Database

The AD biological risk genes were expanded using the STRING database to obtain more candidate drug target genes. The purpose of the STRING database (https://string-db.org/) is to integrate functional interactions related to protein expression by including and arranging predicted protein-protein association-related data (26). We inputted the list of biological AD risk genes selected in the previous steps and set the criterion of 50 interactions. In this way, we were able to increase the number of genes. A larger number of disease-protein networks have greater potential to identify novel therapeutic targets for diseases (35).

### Identification of Drug Target Genes by Using Drugbank and TTD

After completing gene expansion based on PPI information from the STRING database, we conducted an overlapping analysis. The sources of data for the overlapping analysis were the DrugBank database and TTD. Drugbank 5.0 database (www.drugbank.ca) containing around 17,000 associations of drug targets and data on more than 10,000 drug compounds (27). TTD (http://bidd.nus.edu.sg/group/cjttd/) provides information about the 3,101 targets of 34,019 clinically approved and experimental drugs (28). The target genes were used to query the databases according to several parameters, such as drugs with pharmacological activity, human efficacy, and annotations of approved, in clinical trials or experimental drugs.

### Prioritization of Drug Repurposed for AD

All drug targets for AD were confirmed in ClinicalTrials.gov (https://clinicaltrials.gov/) on January 7, 2019 to check whether each drug is under clinical investigation for AD or other diseases. ClinicalTrials.gov is a comprehensive database that documents drugs under clinical investigations in human subjects.

## Results

### AD Risk SNPs and Genes Detected by GWAS

The bioinformatics drug discovery methodologies for AD was shown in **Figure 1**. This study obtained 70 AD associated SNPs from the NHGRI with GWAS *p*-values of less than 1 x 10^-5^ (**Supplementary Table 1**). The selection of SNPs was based on the disease/trait attribute of “Atopic dermatitis.” Next, we expand the number of SNPs by HaploReg v4.1 based on the characteristic of *r^2^* > 0.8 in Asian populations; hence, we obtained 94 AD risk genes (**Supplementary Table 2** **).**

### Functional Annotation of AD Risk Genes

**Figure 2:**
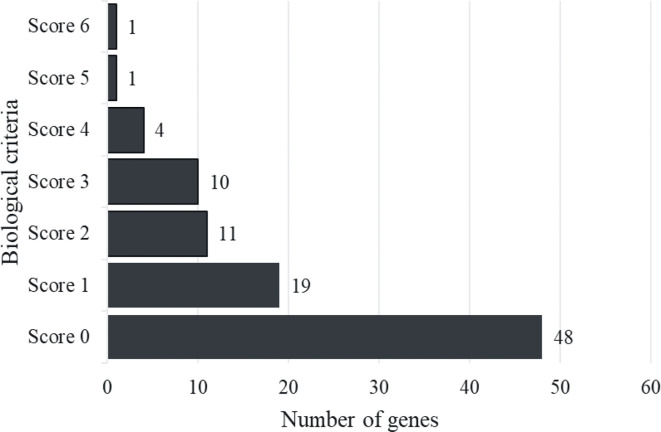
Histogram distribution of gene score. The figure showed that genes with a score of 0 were 48, while those with a score of 1 were 19, and there were 27 genes with total scores ≥ 2 were described as “Biological AD risk genes”.

Six biological functional annotations were applied to prioritize biological AD risk genes. One point was given for each functional annotation. We scored each of the 94 candidate genes by adopting the following six criteria: (1) genes with any missense AD risk variant (*n*=11); (2) *cis*-eQTL genes (*n*=20); (3) genes in any enriched knockout mouse phenotype (*n*=19); (4) genes involved in the enriched GO terms (*n*=26); (5) genes involved in any enriched KEGG pathway (*n*=19); and (6) PID genes (*n*=3) (**Supplementary Table 3**). Thus, each gene earned a score based on the number of criteria fulfilled (score ranging from 0 to 6 for each gene). As shown in **Figure 2**, 48 genes are with a score of 0, 19 genes with a score of 1, and 27 genes with total scores ≥ 2. The 27 genes with a score ≥ 2 were defined as “biological AD risk genes.” As shown in **Table 1**, the top five biological AD risk genes include interleukin 7 receptor (*IL7R*), interleukin 6 receptor (*IL6R*), interleukin 18 receptor 1 (*IL18R1*), interleukin 2 receptor subunit alpha (*IL2RA*), and signal transducer and activator of transcription 3 (*STAT3*).

**Table 1:** Biological atopic dermatitis risk genes.

Gencode Id	Gencode Name	Score	Biological criteria					
			Missense/Nonsense	*Cis*-eQTL	KO mice	PPI	KEGG	PID
ENSG00000168685	*IL7R*	**6**						
ENSG00000160712	*IL6R*	**5**						
ENSG00000115604	*IL18R1*	**4**						
ENSG00000134460	*IL2RA*	**4**						
ENSG00000168610	*STAT3*	**4**						
ENSG00000169194	*IL13*	**4**						
ENSG00000092020	*PPP2R3C*	**3**						
ENSG00000109471	*IL2*	**3**						
ENSG00000113520	*IL4*	**3**						
ENSG00000115594	*IL1R1*	**3**						
ENSG00000134954	*ETS1*	**3**						
ENSG00000138684	*IL21*	**3**						
ENSG00000157456	*CCNB2*	**3**						
ENSG00000172673	*THEMIS*	**3**						
ENSG00000227507	*LTB*	**3**						
ENSG00000258366	*RTEL1*	**3**						
ENSG00000026036	*RTEL1*	**2**						
ENSG00000113522	*RAD50*	**2**						
ENSG00000115602	*IL1RL1*	**2**						
ENSG00000115607	*IL18RAP*	**2**						
ENSG00000168477	*TNXB*	**2**						
ENSG00000182261	*NLRP10*	**2**						
ENSG00000196126	*HLA-DRB1*	**2**						
ENSG00000197114	*ZGPAT*	**2**						
ENSG00000204315	*FKBPL*	**2**						
ENSG00000204525	*HLA-C*	**2**						
ENSG00000213654	*GPSM3*	**2**						

Cis-eQTL, cis-expression quantitative trait locus; KO mice, knockout mouse phenotype; PPI, protein-protein interaction; KEGG, Kyoto Encyclopedia of Genes and Genomes; PID, primary immunodeficiency.

Summary scores obtained from 6 criteria are shown. Filled boxes indicate fulfilled criteria.

### Expansion Network by STRING Database

We utilized the STRING database to integrate publicly accessible sources of information on direct (physical) and indirect (functional) protein-protein interactions (26). Twenty-seven biological AD risk genes were expanded by using the STRING database. Fifty interactions were selected to perform the calculation and expand the number of genes. This study obtained 76 genes as the drug target genes through the STRING database (**Supplementary Table 4**). These drug target genes were used for further analysis.

### Prioritization of Drug Repurposed for AD

**Figure 3:**
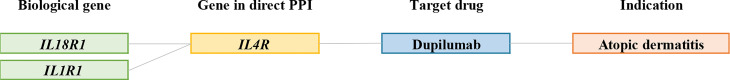
Connections between biological AD risk genes and drugs available for AD. Representative connections between AD biological genes (green); genes in PPIs (yellow); target drugs (blue); indication (orange).

In this step, we obtained 2053 interaction pairs with 76 drug target genes from the curated PPI networking (**Supplementary Table 5**). Furthermore, 76 drug target genes were mapped to DrugBank and TTD. However, not all drug target genes are druggable; only 25 drug target genes were found to bind to 53 drugs based on DrugBank and TTD. The identified target-drug pairs are listed in **Supplementary Table 6**. Among them, dupilumab was clinically approved for AD (**Figure 3**); dupilumab blocks interleukin-4 (*IL-4*) and interleukin-13 (*IL-13*) signaling by binding to interleukin 4 receptor (*IL4R*). IL-4 and IL-13 are the main drivers of the clinical symptoms of AD (36). It is noteworthy that dupilumab is an effective drug for moderate to severe AD (16). As dupilumab was identified from our analysis, the feasibility of gene-based drug repurposing was confirmed.

**Figure 4:**
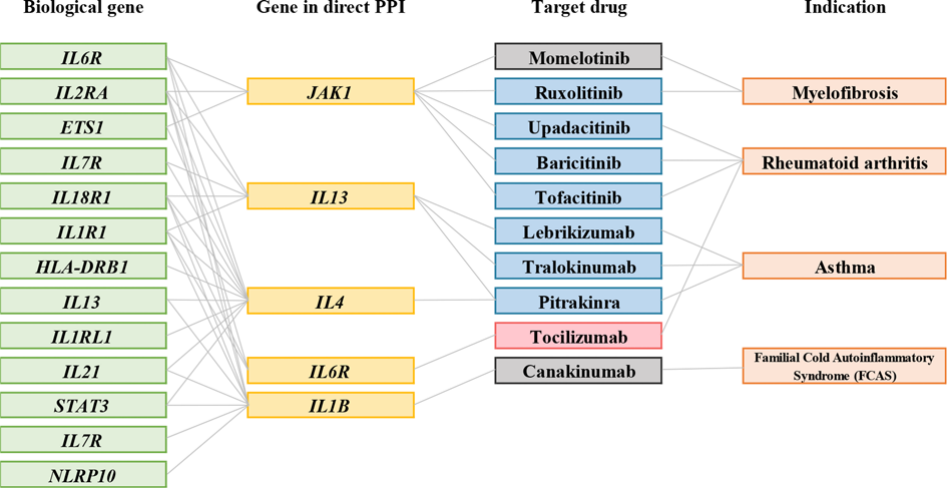
Connections between biological AD risk genes and drugs are potentially useful for AD. Representative connections between AD biological genes (green); genes in PPIs (yellow); target drug for drugs under clinical trial (blue); the drug was supported by case series (pink), and drugs with preclinical data for AD (grey); indication (orange). Grey lines indicate connections. All other biological gene-drug connections are through the PPI network.

In addition, as shown in **Table 2**, ten drugs were found to be potentially useful for AD with clinical or preclinical evidence. Among them, seven drugs were approved for diseases other than AD, including baricitinib (NCT03334422, NCT03334396), tofacitinib (NCT02001181), ruxolitinib (NCT03745651, NCT03745638), upadacitinib (NCT04195698), lebrikizumab (NCT04392154), tralokinumab (NCT03160885), and pitrakinra (NCT00676884). Furthermore, the usage of tocilizumab was supported by case series (42). The effectiveness of momelotinib and canakinumab on AD were supported with animal studies (43, 44). These drugs correspond to 5 gene targets, Janus kinase 1 (*JAK1), IL13, IL4, IL6R*, and *1L1B*, which have the potential to be repurposed for the treatment of AD (**Figure 4**). Examples of repurposed drugs are ruxolitinib and momelotinib, which are approved for myelofibrosis, targeting the *JAK1/JAK2* gene pathway. Ruxolitinib is under clinical investigation for AD in phase III trials (NCT03745651, NCT03745638), while momelitinib has been reported to be repurposed for AD therapy in preclinical investigations (44). Both ruxolitinib and momelitinib are selective inhibitors of *JAK1* and *JAK2* that showed the potential inhibition for proinflammatory cytokine signaling in AD’s pathogenesis (**Table 2**).

**Table 2:** Pharmacological therapies in development for the treatment of Atopic Dermatitis.

**Drug candidate**	**Target**	**Possible mechanism of action on Atopic Dermatitis**	**Disease indication**	**Level of evidence**	**NCT Number/PubMed ID**
**Baricitinib**	*JAK1/JAK2*	Inhibitor of *JAK1* dan *JAK2* mediated signaling in the immunopathology of AD (37)	Rheumatoid arthritis	Phase III completed	NCT03334422, NCT03334396,
**Tofacitinib**	*JAK1/JAK2*	Inhibitor of *JAK1* and *JAK2* inhibits cytokine *IL-4* directly (38).	Rheumatoid arthritis	Phase II completed	NCT02001181
**Tralokinumab**	*1L13*	Inhibitor of *IL-13* by maintaining inflammatory reaction and major skin effects (39).	Asthma	Phase III completed	NCT03160885
**Ruxolitinib**	*JAK1/JAK2*	a selective inhibitor of *JAK1* and *JAK2* potently inhibits proinflammatory cytokine signaling (40)	Myelofibrosis	Phase III ongoing	NCT03745651, NCT03745638
**Upadacitinib**	*JAK1*	a selective inhibitor of *JAK1* inhibited the production of proinflammatory Th2 cytokines such as *IL-4* (41).	Rheumatoid arthritis	Phase III ongoing	NCT04195698
**Lebrikizumab**	*IL13*	Inhibitor of *IL-13* by maintaining inflammatory reaction and major skin effects (39).	Asthma	Phase III ongoing	NCT04392154
**Pitrakinra**	*IL13/IL4*	*IL-4/IL-13* inhibitory activity may reduce inflammation caused by allergens (39).	Asthma	Phase II completed	NCT00676884
**Tocilizumab^#^**	*IL6R*	inhibits *IL-6* binding to soluble *IL6R* (42).	Rheumatoid arthritis	Case series	21962991
Canakinumab*	*IL1B*	*IL-1β* induced *TSLP* production and stimulated keratinocytes (43).	Familial Cold Autoinflammatory Syndrome (FCAS)	–	30937919
Momelotinib*	*JAK1/JAK2*	*JAK1* and *JAK2* inhibitor could reduce inflammatory cytokine expression, including *IL4, IL5, IFN-γ*, and *TSLP* (44).	Myelofibrosis	–	30544712

*Represents under preclinical investigation, ^#^case series.

**Figure 5:**
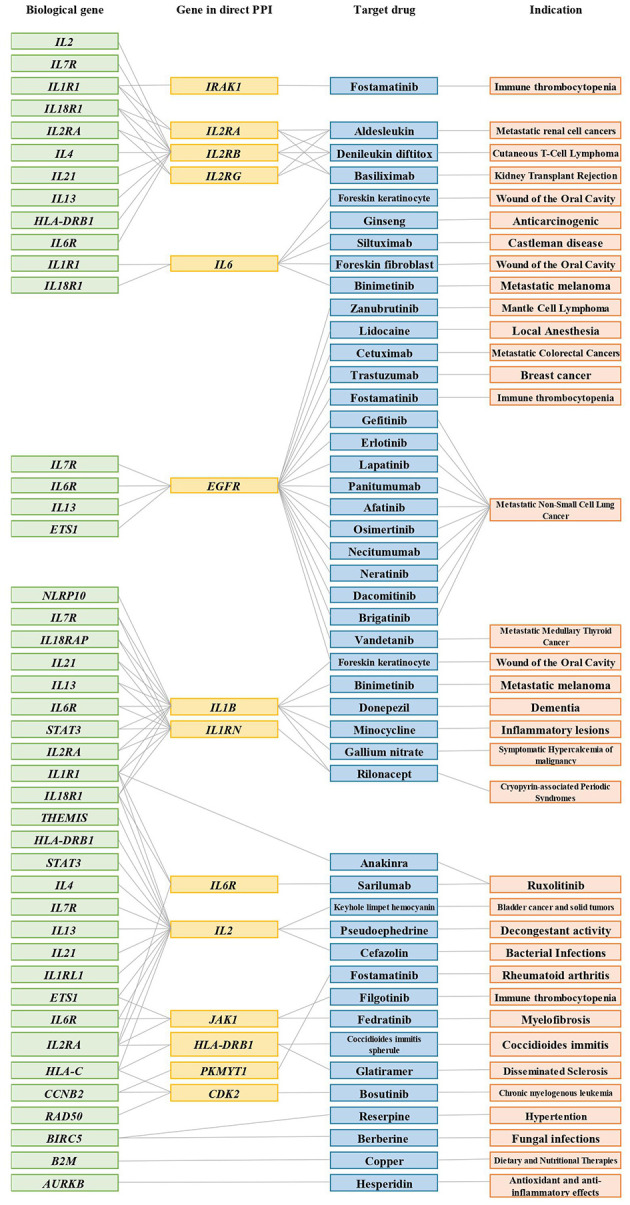
Connections between biological AD risk genes and drugs that were approved for other indications. Representative connections between AD biological genes (green); genes in PPIs (yellow); target drugs (blue); indication (orange). Grey lines indicate connections. Only *IL1R1, BIRC5, B2M*, and *AURKB* are directly connected to a biological gene-drug (anakinra, reserpine, berberine, copper, hesperidin); all other biological gene-drug connections are through the PPI network.

Importantly, we further found 42 unknown anti-AD drugs mapping to 17 drug targets prioritized in our study. The 17 targets are interleukin 1 receptor-associated kinase 1 (*IRAK1*), interleukin 2 receptor subunit alpha (*IL2RA*), interleukin 2 receptor subunit beta (*IL2RB*), interleukin 2 receptor subunit gamma (*IL2RG*), interleukin 6 (*IL6*), epidermal growth factor receptor (*EGFR*), interleukin 1 beta (*IL1B*), interleukin 6 receptor (*IL6R*), interleukin 1 receptor antagonist (*IL1RN*), interleukin 2 (*IL2*), Janus kinase 1 (*JAK1*), major histocompatibility complex, class II, DR beta 1 (*HLA-DRB1*), protein kinase, membrane-associated tyrosine/threonine 1 (*PKMYT1*), cyclin-dependent kinase 2 (*CDK2*), baculoviral IAP repeat-containing 5 (*BIRC5*), beta-2-microglobulin (*B2M*), and aurora kinase B (*AURKB*) (**Figure 5**).

## Discussion

This study focused on repurposing AD drugs based on candidate gene prioritization from the GWAS-identified loci. Using in silico pipelines, we built a scoring system that used six functional annotation for candidate drug prediction. We successfully identified the AD-approved drug, dupilumab, in our study. Furthermore, another ten drugs identified are potentially useful for AD according to preclinical and clinical trial evidence, including baricitinib, tofacitinib, ruxolitinib, upadacitinib, lebrikizumab, tralokinumab, pitrakinra, tocilizumab, momelitinib, and canakinumab, as depicted in **Table 2**. As such, our genetic-driven drug discovery method indicated that a combination of GWAS-based approaches and six functional annotations is able to identify potential candidate drugs for AD effectively.

Among the targets identified, the roles of *IL1B* and *EGFR* in AD have been supported by preclinical evidence. Schwartz et al. reported that anti-IL-1β antibody is able to alleviate dermatitis in the mice model (43). Furthermore, previous studies showed that *EGFR* is involved in the pathogenesis of AD (45). In this study, several drugs are linked to *EGFR*. An example is foreskin keratinocytes, a type of skin cell-cultured as a wound healing and closure. Foreskin keratinocytes are a key component of a number of skin replacements utilized for various indications (46). Keratinocytes are generated from neonatal foreskins and utilized to make a drug called Apligraf, which is a mixture of neonatal foreskin fibroblasts and keratinocytes. Orcel is another skin substitute that combines fibroblasts and keratinocytes produced from the neonatal foreskin, similar to Apligraf (47). In mice model of acute AD, *EGFR* signaling significantly decreases allergen-induced IL-6 production and Th17 responses in the skin, demonstrating that *EGF* has an immunomodulatory impact and is protective in the inflamed skin tissue (45). In addition, our bioinformatic networking analysis specifically identified *JAK* as a potential target for AD. *JAK1* is an upstream regulator of cytokine secretion and immune activation. Hence, the effects of *JAK* inhibitors targeting the multiple immune pathways are a critical mechanism for AD (48). Consistent with previous findings, a preclinical study demonstrated that disrupting *JAK1* signaling is helpful to reduce persistent itch through sensory neurons and immune pathways involving T_H_2 cytokines (49).

Indeed, from the drugs identified through our analysis, five small molecule drugs targeting *JAK* have been supported with clinical and preclinical data in AD. These *JAK* inhibitors are ruxolitinib, baricitinib, upadacitinib, tofacitinib, and momelitinib. *JAK* inhibitors have emerged as a promising therapy option for AD (50). Both oral or topical applications have dramatically improved the clinical outcomes of individuals with insufficient responses to previous medicines in randomized controlled trials (51). In light of the importance of *JAK/STAT* pathways for AD (52), filgotinib and fedratinib were identified as high potential drugs to repurpose for AD in this study. Filgotinib is a selective inhibitor of *JAK1* approved for RA treatment, similar to the marketed drug upadacitinib (53). In phase III randomized controlled trials, upadacitinib was shown to be more effective and well-tolerated than dupilumab for moderate to severe AD (NCT03738397). On the other hand, fedratinib is a selective inhibitor of *JAK2* with a similar mechanism of ruxolitinib (54). Ruxolitinib is a selective *JAK1* and *JAK2* inhibitor. Topical Ruxolitinib Evaluation in Atopic Dermatitis studies (TRuE-AD) demonstrated the safety and efficacy from clinical trials (55).

Another category of drugs identified is monoclonal antibody (mAb) drugs. Four monoclonal antibody drugs (lebrikizumab, tralokinumab, tocilizumab, and canakinumab) were successfully identified as the most promising drug for AD repurposing. Lebrikizumab (NCT04392154) and tralokinumab (NCT03160885) are human monoclonal antibodies targeting *IL-13* under phases III clinical trials for AD. Tocilizumab is *IL-6R* inhibiting monoclonal antibodies and is commonly used in RA. Importantly, functional *IL-6* receptor (*IL6R*) variant have already reported as a risk variant that was associated with persistent AD (56). Thus, blocking of *IL-6* signaling is very likely as a novel therapeutic approach for AD. The other mAb is canakinumab (anti-*IL-1β*) that has been reported as a potential treatment of inflammatory disorders (57).

Although our approaches indicated that the utilization of GWAS data is a potential way of drug mining, there were some limitations. First, by using GWAS data, some SNPs are without biological relevance, and not all drug target genes we identified are directly druggable. Secondly, therapeutic drugs identified through in silico pipelines have not been validated in molecular mechanisms or animal models. Therefore, further investigations are necessary to determine the effects of candidate drugs in clinical applications.

## Conclusion

Drug repurposing offers valuable advantages in the drug development process, such as reduced time and cost, and increased success rates. In the current study, we combine the drug repurposing pipeline with integrative bioinformatics methodologies to identify drugs with novel indications for AD. We found JAK1 inhibitors are particularly important with their involvement in several immune pathways. The results further confirms the feasibility of the application of gene networking and genomic information for drug repurposing.

## Data Availability Statement

The datasets presented in this study can be found in online repositories. The names of the repository/repositories and accession number(s) can be found in the article/**Supplementary Material**.

## Author Contributions 

WA, HW, W-HC, W-PC and W-CC conceptualized, conceived and designed the study. WA and LMI performed the analysis. WA, LMI, W-HC, HW, JT, and W-CC curated the data. WA wrote the original draft. WA, LMI, W-HC, HW, EM, JT, DP, W-PC, and W-CC interpreted the data. WA, LMI, W-HC, HW, EM, JT, DP, W-PC, and W-CC revised the manuscript. W-CC, W-PC, and DP provided the funding. W-PC and W-CC supervised and coordinated this study. All authors have made significant contributions to this study. All authors contributed to the article and approved the submitted version.

## Funding

This work was supported by grants from the Health and welfare surcharge of tobacco products grant (MOHW110-TDU-B-212-144014; MOHW110-TDU-B-212-144020), Ministry of Science and Technology, Taiwan (MOST109-2314-B-038-131 and MOST110-2628-B-038-020), and Taipei Medical University, Taiwan (12310-106079; Yusuke Nakamura Chair Professorship).

## Conflict of Interest

The authors declare that the research was conducted in the absence of any commercial or financial relationships that could be construed as a potential conflict of interest.

## Publisher’s Note

All claims expressed in this article are solely those of the authors and do not necessarily represent those of their affiliated organizations, or those of the publisher, the editors and the reviewers. Any product that may be evaluated in this article, or claim that may be made by its manufacturer, is not guaranteed or endorsed by the publisher.

## Abbreviations

AD, Atopic Dermatitis; AURKB, aurora kinase B; BIRC5, baculoviral IAP repeat containing 5; B2M, beta-2-microglobulin; CDK2, cyclin dependent kinase 2; cis-eQTL, cis-expression quantitative trait loci; EGFR, epidermal growth factor receptor; HLA-DRB1, major histocompatibility complex, class II, DR beta 1; IL-13, interleukin-13; IL-4, interleukin-4; IL-6R, interleukin-6 receptor; IL1R1, interleukin-1 receptor type I; IL1B, interleukin-1 beta; IUIS, international union of immunological societies; IRAK1, interleukin 1 receptor associated kinase; IL2RA, interleukin 2 receptor subunit alpha; IL2RB, interleukin 2 receptor subunit beta; IL2RG, interleukin 2 receptor subunit gamma; IL6, interleukin 6; IL1B, interleukin 1 beta; IL6R, interleukin 6 receptor; IL1RN, interleukin 1 receptor antagonist, IL2, interleukin 2; JAK, Janus Kinase; KEGG, Kyoto encyclopedia of genes and genomes; KOmice, knockout mouse phenotypes; MP, mammalian phenotype ontology; NHGRI, National Human Genome Research Institute; ORA, over-representation analysis; PIDs, primary immunodeficiency’s; PPIs, protein-protein interactions; PKMYT1, membrane associated tyrosine/threonine 1; SNPs, Single nucleotide polymorphisms; TTD, therapeutic target database.
